# Between the snap and the return: a prospective narrative analysis of four voices from inside anterior cruciate ligament reconstruction recovery

**DOI:** 10.1093/ptj/pzag063

**Published:** 2026-06-15

**Authors:** Ramana Piussi, Andreas Ivarsson, Urban Johnson, Kristian Samuelsson, Eric Hamrin Senorski

**Affiliations:** Unit of Physiotherapy, Department of Health and Rehabilitation, Institute of Neuroscience and Physiology, Gothenburg, Sweden; Sahlgrenska Sports Medicine Center, Gothenburg, Sweden; Centre of Research on Welfare Health and Sport (CVHI), Department of Health and Sport, Halmstad University, Halmstad, Sweden; Department of Sport Science and Physical Education, University of Agder, Kristiansand, Norway; Centre of Research on Welfare Health and Sport (CVHI), Department of Health and Sport, Halmstad University, Halmstad, Sweden; Sahlgrenska Sports Medicine Center, Gothenburg, Sweden; Department of Orthopaedics, Institute of Clinical Sciences, Sahlgrenska Academy, University of Gothenburg, Gothenburg, Sweden; Unit of Physiotherapy, Department of Health and Rehabilitation, Institute of Neuroscience and Physiology, Gothenburg, Sweden; Sahlgrenska Sports Medicine Center, Gothenburg, Sweden

**Keywords:** ACL reconstruction, return to sport, narrative inquiry, patient experience

## Abstract

**Importance:**

Anterior cruciate ligament (ACL) injury is a common sports injury with substantial physical and psychological consequences. Qualitative studies have explored patient experiences, but most studies use cross-sectional designs, limiting insight into how experiences evolve throughout rehabilitation.

**Objective:**

The purpose of this study was to explore patients’ experiences of ACL injury and recovery using a prospective, narrative approach, from injury to achievement of self-defined rehabilitation goals.

**Design:**

This was a longitudinal qualitative study with narrative analysis.

**Setting:**

The study was conducted at an outpatient sports rehabilitation clinic in Sweden.

*Participants:* Four adults (ages 20 to 29 years; 2 male, 2 female) undergoing ACL reconstruction were included using consecutive sampling.

**Intervention(s) or Exposure(s):**

Serial 1-to-1 semi-structured interviews were conducted around injury, pre-surgery, early post-surgery, and every 2 months until each participant achieved a rehabilitation goal defined at study entry. Interviews were analyzed using Narrative Oriented Inquiry with attention to story structure, tone, positioning, and context.

**Main Outcome(s) and Measure(s):**

The main outcome were the evolving narratives of participants’ recovery experiences, expressed through first-person interpretative stories.

**Results:**

The longitudinal narratives revealed shifting identities, emotions, and meanings attached to rehabilitation milestones. One participant described moving from helplessness to renewed athletic identity; another portrayed a pragmatic recovery marked by monotony and team reintegration; a third experienced progress until a second ACL injury reframed priorities; and the fourth navigated prolonged knee pain, missed timelines, and gradual acceptance. Across cases, rehabilitation was experienced not as a linear sequence but as an ongoing negotiation of trust, belonging, and confidence.

**Conclusions and Relevance:**

Prospective narrative inquiry illuminated how recovery after ACL reconstruction is lived as a personal, evolving story. For physical therapists, recognizing the temporal dynamics of identity, trust, and confidence may support more individualized rehabilitation, complementing standardized protocols. These findings highlight the importance of attending to both physical and narrative dimensions of ACL recovery in clinical practice.

## Introduction

An anterior cruciate ligament (ACL) injury is a common severe sports-related knee injury,^1^ with an estimated annual incidence of about 68.6 per 100,000 person-years.^2^ Most ACL injuries occur without direct contact, typically during rapid deceleration, landing, or pivoting/cutting with combined valgus/rotational loading.^3^ Treatment options include rehabilitation alone or surgical reconstruction of the ligament, with the 2 most common autograft options being bone–patellar tendon–bone and hamstring tendon (HT).^4^ Rehabilitation after ACL reconstruction is demanding and typically takes at least 1 year before discharge and return to sport.^5^

Upon ACL injury, patients present with pain, uncertainty, and experience a sudden disconnection from their social or athletic environment.^6^ The ACL injury significantly affects both the physical and psychological dimensions of a patient’s life. While quantitative research can quantify outcomes such as function, return to sport, and re-injury rates, qualitative research complements these approaches by exploring how patients experience and interpret recovery.^7^ Qualitative studies including patients with ACL injury have highlighted fear of re-injury, altered trust in the body, and shifts in identity and priorities as central aspects of recovery.^6^^,^^8–12^ From a physical therapy perspective, such experiences are clinically relevant because they shape how patients engage with rehabilitation, interpret progress, and approach return to sport. Thus, these findings suggest that ACL rehabilitation is not solely a biomechanical process, but also a psychological and social transition.

Recovery after ACL injury and reconstruction unfolds over time. Emotional responses, expectations, and perspectives may shift across different phases of rehabilitation, from injury and surgery to return to sport. A prospective narrative design allows exploration of how patients interpret and re-interpret their experiences as rehabilitation progresses, capturing transitional moments and evolving perspectives, and adding insight into how emotional responses, expectations, and perspectives are shaped across the course of rehabilitation.^13^ Unlike longitudinal thematic approaches, narrative inquiry also examines how experiences are storied, through shifts in eg, stance, emotion, and interpretation across phases of recovery. Such knowledge is important for clinicians, as it can inform more individualized approaches to rehabilitation and communication with patients.

The aim of this study was to explore patients’ experiences of ACL injury, from the time of injury to the time in which patients achieve their respective rehabilitation goal, using a prospective design.

## Method

To explore patients’ experiences of ACL injury, from the time of injury to the time in which patients achieve their respective rehabilitation goal, a sample of 4 patients was selected,^14^ and a longitudinal qualitative study was conducted in parallel with each patient’s rehabilitation. The study was approved by the National Ethical Review Board (dnr: 2024-08724-01) and conducted in accordance with the Declaration of Helsinki. All participants provided oral recorded informed consent prior to participation. This study was reported according to the Standards for Reporting Qualitative Research checklist.^15^

**Figure 1:**
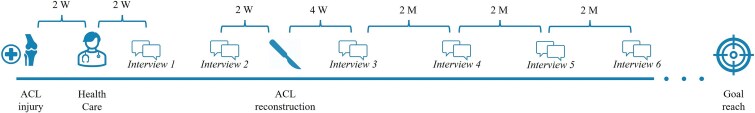
Timeline of Interviews. Health care refers to the first visit by a physical therapist at a sports rehabilitation clinic, and the start of the rehabilitation process. Interviews continued at approximately 2-month intervals until participants achieved their individual rehabilitation goal. Therefore, number of interviews varied between participants. ACL = anterior cruciate ligament; m = months; w = weeks.

Participants were recruited using consecutive sampling. Four consecutive patients, 2 male patients and 2 female patients, aged 20 to 29 years (result section), who sought care at a sports rehabilitation clinic in Sweden within 2 weeks of knee injury and presented with clinical signs of ACL rupture (ie, increased knee laxity, pain, stiffness, and reduced trust in the knee) were invited to participate. Upon inclusion, several individual semi-structured interviews were scheduled with each participant according to the following timeline: time of injury (within 2 weeks of first appointment with the health care provider), pre-surgery (within 2 weeks before planned ACL reconstruction), post-surgery (within 4 weeks after ACL reconstruction), and then every second month until the patient reached their pre-specified goal with rehabilitation, which was defined during the first interview (Figure 1).

Interviews were semi-structured (Suppl. Material), and follow-up probe questions were used throughout the interviews to encourage elaboration and clarify participants’ experiences. The time of each interview and the 2-month interval between them were selected by the authors to reflect key stages in the ACL rehabilitation process. Two interview guides (Suppl. Table 1 and Suppl. Table 2) were drafted: 1 for the first interview, and 1 for the follow-up interviews. Guides were drafted through communication among the authors’ team and screening of the literature on the subject. Guides were not pilot tested. Interviews were performed electronically via the Zoom web-based application (Zoom Video Communications, Inc. San José, CA, USA). All interviews were conducted by the first author (R.P.), audio recorded and transcribed verbatim. No other person beside the first author and the participant were present at each interview, and no field notes were taken during interviews. The total recorded time for all interviews was 427 minutes.

After completion of data collection, the transcripts were analysed using Narrative Oriented Inquiry (NOI) as proposed by Hiles, Čermák, and Chrz.^16^ The NOI is grounded in a constructivist—interpretivist paradigm in which reality and knowledge are understood as socially and narratively constructed, contextual, and co-produced through discourse and lived experience.^17^^,^^18^ Accordingly, narratives were treated as situated accounts that convey both events and the teller’s interpretation of what matters, expressed through how the story is told, including emphasis, reflection, and emotion.^16^^,^^19^ This stance informed study design and analysis by prioritizing the temporal development of patients’ stories and attending to meaning-making, narrative positioning, tone, and contextual framing rather than solely factual chronology.

In this study, authors followed the analytical framework and principles described by Hiles, Čermák and Chrz,^16^ and supplemented the narrative understanding with insights from Herman and Vervaeck’s Handbook of Narrative Analysis.^20^

### Analysis

1) The research question was clarified, and patient narratives were first divided into segments; with each segment representing a distinct and meaningful episode (eg, injury, surgery, a rehabilitation milestone, or a setback) in the patient’s story.
2) Sjuzet was distinguished from fabula. The sjuzet refers to the situated and occasioned telling of the story, which includes emphasis, commentary, and reflection. In other words, it is the “way” the story is told. In contrast, the fabula is the basic outline of the events as they occurred (or might have occurred). The fabula is described as bounded, because changes in it (even details) will change the story being told.

Thereafter, 3 additional levels of narrative analysis were then applied^20^:

3) Narrative positioning: who is the narrator and where do they stand?
4) Narrative tone and emotion
5) Contextual framing: social and cultural resources

The first author (R.P.) conducted the primary narrative analyses. Throughout the analytic process, interpretations regarding narrative tone, positioning, contextual framing, and overall narrative construction were continuously discussed with the senior author (E.H.S.). Analytic decisions and narrative representations were critically reviewed after analysis of each patient with the second author (A.I.). Interview transcripts and analysis were managed and organized using Microsoft Word and Microsoft Excel (Microsoft Corporation, Redmond, WA, USA).

An illustrative example of the analytic process is presented in Table 1.

**Table 1:** Sample of narrative analysis

Interview text	Fabula	Sjuzet	Narrative positioning	Narrative tone and emotion	Contextual framing
“Before the operation, I went to internet and saw how people felt… a lot of people said that this is really like a mental game… sometimes very hard. But what I noticed is that it is also kind of rewarding this healing process. Cause every single day, I can see some kind of progress… every day you can do something new… it really gives you hope and it kind of makes you excited to be able to do things that you couldn’t do yesterday…” Patient 1, “Marie.”	After surgery, rehabilitation progressed faster and more positively than expected; daily improvements were experienced.	Contrast structure (“people said… but I noticed…”); repetition of “every day”; emphasis on novelty and reward; evaluative language (“really rewarding,” “gives you hope”).	Narrator positions herself as an active observer of her own recovery; she stands inside the process but slightly above it, evaluating and comparing expectation vs reality.	Hopeful, energized, positively surprised; emotionally upward movement.	Early post-operative phase; comparison with online narratives; framed as discovery that rehab is not only struggle but growth.
“That was my first training after finishing physical therapy… for about an hour I was able to train normally… but at some point… I just stepped on the leg and then it gave away. It hurt quite a lot and after that it got swollen… At first I was so sure that it was my ACL again… it felt super unfair. I felt I did everything that I needed to do… and now I really don’t think I will be playing rugby this season… I just really hope that maybe in a year or in the future I will be able to go back…” Patient 3, “Lucy.”	After completing rehab and returning to rugby training, the knee gave way during training; swelling and pain followed; uncertainty about damage; possible re-injury.	Gradual build-up (“first training… for about an hour…”); abrupt turning point (“but at some point…”); evaluative intensifiers (“very, very sad,” “super unfair”); shift from certainty to uncertainty; forward-looking speculation.	Narrator positions herself first as prepared and compliant (“did everything needed”), then as wronged by circumstances; she stands in a reflective present, looking back at the event with altered perspective.	Initially catastrophic and unfair; then reflective, cautious, and resigned; emotional movement from shock to sadness to reframing.	Occurs after declared goal achievement; transition from rehabilitation setting to uncontrolled sport context; framed against the expectation of successful return.

In the final step of the NOI, an interpretative synthesis was conducted to integrate insights from the 5 preceding levels (fabula, sjuzet, positioning, tone/emotion, and contextual framing) into a coherent understanding of each patient’s overall narrative. This involved revisiting the complete interview set to trace how meaning, identity, and emotional stance evolved over time, with attention to narrative shifts and consistencies.^20^

These analytic steps were performed separately for each included patient, with one patient’s interview set analysed at a time. This process was used to construct, for each patient, a short description of key events and narrative levels, followed by a first-person short story told in the voice of a fictionalized character. Each story integrated the character’s background, injury, decision-making, surgery, rehabilitation, and recovery alongside the key findings of the NOI. As this study used NOI, the concept of data saturation was not applicable. Instead, data sufficiency was considered in terms of information power**,** that is, whether the sample was expected to provide sufficiently rich and relevant information for the study aim. Given the narrow aim, the specificity of the sample, and the depth afforded by serial interviews across rehabilitation, 4 participants were deemed to provide sufficient information power for an in-depth case-based narrative analysis.^21^

### Reflexivity

Reflexivity involves ongoing critical reflection on how researchers’ perspectives and assumptions influence interpretation. To improve reflexivity, author team credentials are disclosed: the principal investigator (R.P.) is a male physical therapist with nearly 10 years of experience of sports injury rehabilitation. R.P. has extensive experience of qualitative studies, and conducted all interviews and the primary narrative analyses in this study. Both the second (A.I.) and third (U.J.) authors are male researchers with PhDs in psychology (both professors) with extensive experience in the sports psychology research field. Both A.I. and U.J. also contributed to the interpretation of narratives and reflexive discussions during the analytic process, and both have previous experience of qualitative research, including narrative-oriented interpretation of experiences in sport and rehabilitation-related contexts. In addition, author A.I. is a sports psychology consultant. The fourth author (K.S.) is an orthopedic surgeon (professor), who is a former elite Judoka and has had an ACL injury on both knees, and who has extensive experience of clinical treatment and research in the field of ACL injury. The senior author (E.H.S.) is a male physical therapist (professor) who has over 15 years of experience in clinical and research of sport injury rehabilitation. E.H.S. has extensive experience of qualitative research, and provided senior oversight throughout the narrative analytic process.

No relationship was present with the included patients prior to study commencement, and no bias or assumptions were communicated to included patients during the study period.

### Trustworthiness

Trustworthiness was considered using the criteria of credibility, transferability, dependability, and confirmability.^22^ Credibility was supported by serial interviews over time, use of an explicit analytic procedure (segmentation into episodes; fabula/sjuzet distinction; narrative positioning; tone/emotion; contextual framing; and interpretative synthesis), and iterative peer debriefing within the author team during analysis and narrative construction. Transferability was supported with the description of the study context, participant characteristics, and interview timing to enable readers to judge relevance to other settings. Dependability was enhanced by applying the same analytic steps to each participant and by presenting an illustrative example of the analytic process (Table 1). Confirmability was supported through reflexive discussions among authors to challenge interpretations and ensure that the fictionalized first-person stories remained grounded in the interview material.

Transcripts were not returned to participants as this method has been criticized since participant feedback may reflect later reinterpretations rather than the accounts captured at interview.^23^

## Results

Four patients, 2 male and 2 female, were included in this study (Table 2). For Patients 1 to 3, surgery were performed with HT autografts, and for Patient 4 with a patella tendon autograft.

**Table 2:** Demographic of included patients

Patient^a^	Age at injury	Sport	Sport level	Surgery	Achieved goal	Number of interviews
1	20	Track & field	Competitive sub-elite	Early 2023	May 2024	9
2	20	Handball	Recreational/amateur	Early 2023	May 2024	9
3	29	Rugby	Recreational/amateur	Early 2023	February 2024	8
4	24	Floorball	Competitive sub-elite	Early 2023	November 2024	12

^a^ To ensure patient’s confidentiality, patient sex and exact dates of surgery are not provided.

### Patient 1: Fictionalized as “Marie,” track & field athlete

**Figure 2:**
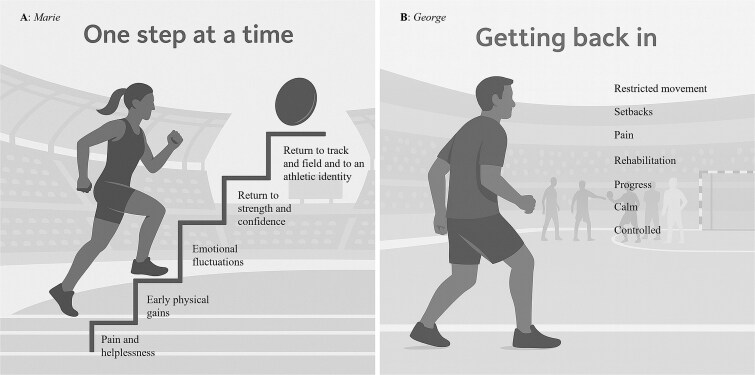
Panel A (Marie) and B (George). Panel A Shows Marie’s Recovery as a Step-by-Step Process, Moving from Pain and Helplessness to Renewed Confidence, Strength, and Return to her Athletic Identity. Panel B Shows George’s Recovery as a Steady Return toward Sport, with Progress Shaped by Pain, Setbacks, Restricted Movement, and Gradual re-Entry into Handball. Picture Created with ChatGPT, Version 5.

Marie’s story begins with a knee trauma (2018) which she initially managed non-operatively on her own. She avoided medical care, continued her athletic life, and adapted to increasing limitations. Years later, persistent instability, pain and a new knee trauma (for which she was included in the present study) led her to seek medical care, and an ACL reconstruction in 2023. The narrative unfolds through initial pain and helplessness, early physical gains, emotional fluctuations, and the gradual return to strength, confidence, and identity as an athlete (Figure 2, panel A). Ultimately, she returned to activities she had not done for years, including jumping and strength training. Marie’s story is told chronologically, but not strictly linearly. She uses emotionally varied language: casual humor, vivid physical detail, and performance metaphors. The storytelling becomes more confident and fluid over time. Marie positions herself as an independent, self-managing athlete who has “pushed through” pain. As the story progresses, she repositions herself as a disciplined patient who takes responsibility for her body. She transitions from an “injured identity” to an “athlete” with renewed competitive motivation. The tone moves from sadness and frustration to hope, curiosity, joy and pride. In the early interviews the tone is heavy, hesitant and ironic, while in the middle phase is reflective and pragmatic, to finish as confident and playful in the last interviews. Marie’s story relies on her sport identity, the experience with the health care system, where she receives a lot of support from her physical therapists, and her friend network.

*Interpretative, short first-person story for patient 1. Title: “one step at a time”.*

For years, I managed my knee with tape, willpower, and rest. It first twisted while skiing, then collapsed again during a jump. After that, pain invaded daily life: walking, squatting, even jogging. So I finally sought care and scheduled ACL reconstruction.

I thought I was ready. I was not. The first week after surgery was brutal: dizziness from pain, a leg that would not respond, bruising that spread down my calf. I felt I had lost the body I knew. Rehabilitation began with tiny movements. My physical therapist, calm, persistent, marked each gain. Ten more degrees. Off crutches. Walking again. The first time I did a bridge with both feet, I nearly cried. It felt like mine again. Progress was slow and fear persisted, especially jumping, but gradually I rebuilt trust. Gym sessions turned from rehabilitation into training. My thigh grew stronger, exercises resembled my old routines, and for the first time I pushed myself. Hop tests became a turning point. What I once avoided, I cleared with a landing that felt like flight: small, but pain free (quote 1). That moment told me I was back, not finished, but myself again.

Quote 1: “It [the knee] feels more like a friend than an enemy these days… So now it is really like this… I am starting from a point where my foot barely leaves the ground, to actually being able to get a little air between my foot and the ground… I am really taking it one step at a time.”

Late rehabilitation brought speed, strength, and throws that reminded me of my sport. Muscle memory returned, and the fear that dominated early recovery began to fade. Training was no longer dictated by my knee, but by me. People ask what made the difference. Surgery mattered, but it was daily discipline, steady trust-building, and the support of those around me, especially my physical therapist. My knee is not perfect. But it is mine again. And I have never felt stronger.

### Patient 2: Fictionalized as George, handball player

George sustained an ACL injury during a handball match, following an awkward landing. Post-surgery, George went through expected phases: pain, restricted movement, early mobility, return to gym and sport-specific training, and a gradual increase in function. He experienced a significant setback when he overtrained and felt to have injured his hamstring (painful pulling in the back of the tight), which delayed full return to sport. Despite the setback, he maintained steady progress with rehabilitation and eventually returned to handball competition. George’s narrative is told in an unfiltered, conversational voice. It is often spontaneous, filled with pauses, fillers, and reflective corrections (Figure 2, panel B). The story follows a chronological progression, but with frequent retrospective reflection. He uses humor lightly and often understates emotional moments. George positions himself as a determined, practical athlete involved in his sport. As the narrative progresses, he repositions from “injured athlete on the sidelines” to “team member doing modified participation” to “athlete regaining full function.” He never fully identifies as a “patient,” instead, he leans into an identity of someone “on the way back.” George’s tone is calm and controlled through the interviews. He rarely dramatizes his emotions. His sadness, fear, or motivation is more often implied than emphasized. Fear of re-injury appears repeatedly, but never spirals: it is acknowledged, managed, and contained. Motivation dips are framed as understandable and temporary, not as an existential crisis. George’s narrative draws from different social and cultural narratives such as sport identity with stoicism, team belonging and expert-led trust: he places full trust in his physical therapist to validate his progress. He casually acknowledges mental aspects like motivation, identity, and fear. His narrative resists “heroism” and leans instead into a quiet resilience.

*Interpretative, short first-person story for patient 2. Title: “getting back in”.*

I remember the pop. Not loud, not dramatic, just a small sound that changed everything. Mid-air for a shot, landed off, knee folded. I knew. Friends had torn their ACLs; I felt it in my bones. The hospital was uncertain, but I was not. It was confirmed soon after: ACL tear, surgery, rehabilitation.

It hit me hard. Sport was life: handball was my structure, my people. The thought of being out felt like a hole I could not fill (quote 2).

Quote 2: “Um… right now it really sucks. I mean, I cannot train or do anything like that, and it has… it [handball] used to be a very big part of my life. Or well, I can train and stuff, but I cannot really take part in handball practice, you know, it does not work like that, I cannot join in. And that is very, very hard mentally, because it [handball] was such a big part of my life. So it is really hard.”

The first weeks after reconstruction were rough: pain, stiffness, swelling, even getting into a car was a struggle. When rehab started, I was frustrated by how long everything took. One week progress, the next week stuck. My physical therapist kept me grounded, especially after I overtrained and nearly tore my hamstring. He reset my plan and pushed me to stick with it. Motivation dipped. Work and coaching got in the way, and I slacked off. But I never quit completely. The middle stretch of rehab was the hardest: flat, repetitive, progress almost invisible. What kept me going was being with the team. Even off the court, being part of the group reminded me of who I was. Jogging was a breakthrough. Then agility, passing, non-contact drills. Slowly, joy came back, and I started trusting my knee again. Still, fear lingered, especially with jumping. A whisper in my head: what if it snaps again?

Now I’m back on the pitch. Not at 100% yet, but close: training, sprinting, cutting, passing. Confidence is still building. Looking back, I see a slow story, full of dips and climbs. Hours alone in the gym, teammates making space for me, and a guy who did not quit. Not because it was easy, but because he wanted back in.

### Patient 3: Fictionalized as Lucy, rugby player

**Figure 3:**
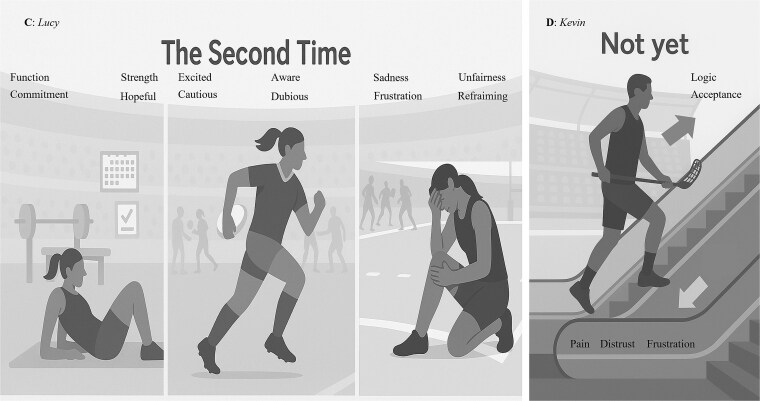
Panel C (Lucy) and D (Kevin). Panel C: graphic representation of Lucy’s story “the second time.” Lucy is represented as a rugby player doing rehabilitation (first slide), returning to rugby (second slide) and injuring her ACL a second time (third slide). Important narrative levels are placed above her. Panel D: brief graphic representation of Kevin’s story “not yet.” Kevin is represented as a floorball player trying to climb a downward moving escalator. Important narrative levels are placed around him.

Lucy tore her ACL during a non-contact situation at a rugby training. She chose to have surgery and embarked on a structured 1-year rehabilitation process, supported by a physical therapist she trusted. Lucy experienced strong physical progress: regained function, resumed strength training, started running and jumping, and overcame initial fears. Lucy returned to rugby training 1 year after reconstruction, but her knee gave away in the first session, and she sustained a second ipsilateral ACL injury. Lucy tells her story in a clear, reflective and emotionally layered way. Her narrative moves chronologically, but with a dual awareness of what she hoped for and what happened. She frequently contrasts before and after moments eg, before ACL reconstruction versus after, to make sense of shifts in her journey. Over time, her narrative style matures: early optimism becomes tempered with caution; eventual disappointment brings philosophical depth. Lucy positions herself as disciplined and committed, reflective, emotionally engaged and trusting towards the physical therapist’s knowledge. She is not positioned as a victim, despite the outcome. Instead, she frames herself as someone willing to adapt, even if it is emotionally difficult. Lucy has a rich and dynamic emotional landscape. In the early interviews she is worried but hopeful. During rehabilitation she is motivated, positive, and surprised by her progress. In the last interviews she is excited but cautious, and finally sadness and frustration take over after the ACL re-injury (Figure 3, panel C). Lucy’s narrative is embedded in several important contexts, such as athletic identity, where rugby is more than a sport for her, and is tied to joy and personal freedom. In addition, Lucy reshapes her physical self-concept, moving from feeling invincible to feeling vulnerable, and goes through a psychological recalibration where health becomes more important than rugby.

*Interpretative, short first-person story for patient 3. Title: “the second time”.*

I used to think my body was bulletproof, the quiet, unconscious kind of trust that comes from years of showing up and relying on it (quote 3).

Quote 3: “Eh well, eh like I said it is my first... injury ever… so it really eh… change the way that I see my body. I really thought it is kinda invincible before.”

I played rugby hard: pivoting, tackling, getting up, and moving on. Then 1 day it did not. A wrong step in a drill, no contact, no crash. Just a deep, unfamiliar pain. At first they said it might be fine. Weeks passed and my knee still felt off. MRI: ACL tear. Surgery recommended. I hesitated, not because I feared surgery but because I feared what it meant: time away from rugby, from the part of myself I did not know how to live without. I chose surgery because I wanted to come back strong. The operation went well. Rehabilitation began. I threw myself into it: disciplined, structured. I took notes, tracked progress. It felt like a mission. And it worked. Gradually, the basics returned: bend, walk, strength, balance. My physical therapist said I was ahead of schedule. We tested strength, hop distance, and symmetry. I hit the numbers. Still, a long, quieter phase followed: progress slowed to increments. The gym routine became repetitive; gains were measured in small percentages: a bit more power, a touch more control. I practiced every day, ticking off checkpoints, but something kept scanning for weakness. The numbers said I was ready; I wanted to believe them. Mostly I did. Mostly.

Outside the clinic, doubt lingered. I filmed myself, checked angles, compared left and right. Running on grass, changing direction, the body felt alert in a new way. It was not panic. It was awareness: chaos on the field could expose what the gym hid. You ca not plan every bounce or foot placement in rugby.

Twelve months in, I was back. Team training, taped, warmed up, mentally prepared. It was supposed to be a celebration. Forty minutes into drills and contact, my knee went. No collision, just a slip, a shift, and a betrayal. I lay there and knew. Later, tears and nausea: I had done everything “right.” How could this happen again? I told the expected lines about risk and no guarantees, but inside I felt deceived. Not by my physical therapist or tests, but by the assumption that doing it right ensured it would go right (quote 4).

Quote 4: “At first it was really really sad. I felt it was kinda unfair, because I really did everything that I needed to do in order to… I did what the knee needed to be… be good.”

The weeks afterward were fog: no rehab talk, no rugby. I refused immediate decisions. I walked, worked, let myself be sad. Slowly the edge softened. I reframed things: maybe rugby would not be the whole story anymore; maybe invincibility was a myth, and that was not the end of meaning. I do not know what comes next. More scans, another surgery, or a different path. What I do know is this second rupture taught me something the first did not: control is not protection, and structure, while helpful, offers no guarantees. Sometimes doing everything right still asks you to let go, and letting go does not have to mean giving up.

### Patient 4: Fictionalized as Kevin, floorball player

Kevin sustained a non-contact ACL injury during a football match and chose ACL reconstruction with the goal of returning to competitive floorball. At first, he imagined a textbook recovery: disciplined rehabilitation, steady progress, and a return to sport within a year. But things did not go as planned. Persistent patellar pain emerged partway through rehabilitation, stalling progress and disrupting timelines (Figure 3, panel D). Kevin’s narrative is structured, controlled, and methodical. He speaks with a calm, rational tone, using clear sequencing and practical language, with no dramatics and no exaggeration. He revisits the same moments from different angles, as if trying to make sense of delay through logic. His style is measured and reflective, sometimes tinged with quiet irony. He positions himself as disciplined and realistic, someone who takes responsibility and adapts rather than complains. Over time, his narrative shifts from hopeful planning to mature acceptance. His physical therapist is a steady collaborator, not a central character. The emotional tone is low-key and contained: frustration is acknowledged but rarely expressed intensely. Kevin’s narrative unfolds through themes of time, control, adaptation, and identity, and is shaped by broader life transitions, including a desire for freedom and a return to sport on his own terms. The narrative for Kevin is shaped by several key contextual structures. He is an active person with meaningful ties to his sport, team culture, and has a strong athletic identity. He is in a transitory situation in life: Post-university adulthood and the desire to embrace personal freedom (eg, travel) influence how he perceives restriction and recovery. Over time, Kevin’s narrative shifts from hopeful planning to mature acceptance. The ACL injury challenged his sense of physical control but also invited deeper reflection on how life does not always bend to willpower. Beneath the calm and structure lies a quiet negotiation with time, identity, and the need to redefine what it means to return.

*Interpretative, short first-person story for patient 4. Title: “not yet.”*

I tore my ACL and made a plan: surgery, rehabilitation, return in a year, maybe 10 months if things went well. I am realistic, but I like structure, and I do not mind the work. The surgery went smoothly, rehabilitation started strong, strength returned. For a while I thought I might even beat the 12-month mark. Then the pain started. It was not sharp, just a persistent ache in the front of my knee. Squats, steps, lunges, and it was always there. Rest helped briefly, but every push brought it back. We adjusted exercises, tweaked loads, followed the book. Still, the pain lingered. Ten months passed. Then 12. Then 15. I was still in the gym, still modifying, but not sprinting, not jumping, not cutting. The neat timeline I had made became a joke.

It all felt flat. Not stuck, exactly, but slower. I trained 3 times a week, but momentum was missing. Each flare-up sent me back into the loop: push, pain, pull back. Repeat (quote 5).

Quote 5:“I guess it is that when… the plateau… like when the pace of rehab starts to level off… that probably came a earlier than I had expected… Right now, if it keeps progressing at the same rate, I find it hard to see that I’ll be able to play [floorball] … I notice that it hurts less, but it doesn’t hurt so much less that it will be completely gone in 6 months….”

I knew more about rehabilitation than I ever expected. Enough to second-guess myself. Should I rest more? Push more? Wait? I felt lost. The goal was still there, but the timeline was gone. Training became steady maintenance, no milestones, no fireworks. The frustration built quietly. I did not quit, but I stopped pretending it would be simple. I trained, I adapted, and eventually I let go of deadlines. Let go of the picture of a clean one-year comeback. It felt like walking up a down escalator - always working, never quite arriving.

Now, I’m back on the floorball court. I can play full matches, the very thing I visualized through endless sessions. But it is not as I imagined. The knee works, but differently. I feel tightness, hesitations, an awareness I never carried before. I do not trust it completely, not like before. So yes, I returned. Fully. But not to the version of sport, or the version of me, I thought I was chasing.

## Discussion

This prospective narrative study illustrates that recovery after ACL injury and reconstruction is not a uniform pathway but a lived, evolving story. The prospective design and the narrative analysis revealed changes in emotional stance and attitudes, priorities and self-understanding, which emphasize the importance of rehabilitation not only as a physical process but also as an ongoing negotiation of trust, identity, and belonging. Our findings suggest that key clinical periods (from early rehabilitation to return-to-sport preparation and return attempts) are also narrative turning points where clinicians may support confidence, identity, and belonging, alongside physical recovery. Table 3 provides a cross-case synthesis of phase-by-phase narrative shifts and proposed clinician-oriented action points.

**Table 3:** Cross-case synthesis of phase-by-phase narrative shifts and proposed clinician-oriented action points^a^

Phase	Cross-case narrative shifts	Proposed clinical action points
Injury and early contact	Uncertainty; disruption of identity/routine; early body distrust; reduced belonging to sport/social context	Validate uncertainty; provide clear roadmap; support continued connection to sport/community where feasible
Early rehabilitation	Progress often fuels hope; vigilance about symptoms may coexist	Reinforce short-term gains; normalize emotional variability; support self-efficacy
Mid-rehabilitation	Rehabilitation becomes routine; motivation can fluctuate; sport identity can feel “on hold”	Address adherence dips; maintain motivation; support belonging/identity while sport participation is limited
RTS preparation	Confidence and fear often co-exist; “trust” becomes central	Graded exposure; normalize ambivalence; align physical preparedness with perceived confidence/readiness
RTS	Excitement + vigilance; heightened meaning attached to first sessions	Plan re-entry; set boundaries and gradual progression; prepare for normal physical and/or psychological fluctuations
Setbacks (if applicable)	Abrupt meaning shift (loss of control/unfairness); reorientation of goals	Support meaning making; prevent disengagement; reframe setbacks within trajectory

^a^ RTS = return to sport.

Prior qualitative ACL research has described rehabilitation as demanding,^24^^,^^25^ with fluctuating motivation,^26^^,^^27^ fear of re-injury,^12^^,^^27^^,^^28^ and uncertainty about return to sport^28^ as common themes. Building on this work, the present prospective narrative design highlights how such experiences evolve across rehabilitation phases rather than being confined to a single moment.^29^^,^^30^ The cross-case synthesis proposed in Table 3, suggests that recovery after ACL reconstruction involves a continuous negotiations of body trust and belonging, where patients shift between hope, vigilance, confidence, and uncertainty as rehabilitation tasks become more sport-specific. These shifts were reflected not only in what patients described, but in how experiences were told, interpreted and re-interpreted across time.

The NOI supported analysis of how stories were told over time, including changes in narrative stance, emotion, and contextual framing.^20^^,^^31^ Important features of NOI are transparency, inclusivity and the use of multiple interpretive perspectives combined with reflexive examination of analytic decisions.^20^^,^^31^ This approach was well suited to a clinical setting because it preserves narrative complexity and patient voice but offers at the same time a structured interpretive framework.^20^^,^^31^

### Clinical implications

Viewed alongside stage-based rehabilitation models after ACL reconstruction that grossly divide recovery into an early, mid- and late stage,^32^^,^^33^ findings from the present study suggest that patients’ clinical needs shift across recovery phases rather than remain stable throughout rehabilitation. In the early phase, when uncertainty, disruption, and reduced trust in the knee were prominent in the narratives, clinicians may support patients through acknowledgement of uncertainty and a clear rehabilitation roadmap. In the middle phase, when rehabilitation became repetitive and progress less visible, attention to motivation, belonging, and shifts in self-understanding may be clinically relevant. If setbacks occur, early support may focus on meaning, goal reorientation, and continued engagement with rehabilitation. In the later phase, particularly during return-to-sport preparation and return attempts, confidence and vigilance can co-existed. At this stage, graded re-entry alone may not be sufficient; explicit dialogue about trust, expectations, and normal emotional fluctuations may also help patients navigate the transition. Clear and honest communication should always be a pillar of the clinical interactions between clinicians and patients.^34^

### Limitations

In terms of credibility, findings reflect interpretations derived from serial interviews and narrative construction; participants did not review the fictionalized stories and alternative readings of the material remain possible. Regarding transferability, the sample included 4 athletes treated with ACL reconstruction within a single-country context; therefore, applicability to non-athletes, other health care contexts, and patients treated without reconstruction should be considered with caution. With respect to dependability, the study did not standardize or track surgical details, rehabilitation content, rehabilitation dose, or return-to-sport testing approaches; these contextual factors may have influenced experiences across cases. Finally, confirmability remains constrained by the interpretive nature of NOI; reflexive and collaborative analysis was used to enhance transparency, but researcher judgment can possibly always influence narrative synthesis. These limitations should be considered when interpreting the findings, which are intended to illuminate processes and meanings rather than to generalize to all patients with ACL injury.

## Conclusion

This study illustrates that recovery after ACL injury and reconstruction is not experienced as a fixed sequence of milestones but as a shifting, individual journey, unique for each patient. These findings extend previous cross-sectional qualitative research and add what patients experience and how experiences change. Rehabilitation is lived as an ongoing negotiation of trust, belonging, and confidence. While clinical milestones and rehabilitation programs may follow standardized timelines, the meaning patients attach to them is deeply personal.

## Supplementary Material

PTJ-2025-0967_R2_Supplementary_Material_Qualitative_Prospective_PTJ_pzag063

## Data Availability

Data is available from the corresponding author upon reasonable request.
