# Supplementary material for: Between the snap and the return: a prospective narrative analysis of four voices from inside anterior cruciate ligament reconstruction recovery
Source: Phys Ther. 2026 Jun 15;106(7):pzag063. doi: 10.1093/ptj/pzag063 (PMC13403183; doi:10.1093/ptj/pzag063)
Supplement: PTJ-2025-0967_R2_Supplementary_Material_Qualitative_Prospective_PTJ_pzag063 [file ptj-2025-0967_r2_supplementary_material_qualitative_prospective_ptj_pzag063.pdf]

Supplementary Material

Table 1: interview guide for first interview
Can you tell me about the occasion when you injured your ACL?
How do you feel right now?
What does this injury mean to you? What consequences are you experiencing from being injured?
What thoughts do you have about your knee?
What thoughts do you have about the rehabilitation?
What do you think will happen?
What is your goal?
What do you think will be required to achieve your goal?
What could potentially hinder you from achieving your goal?
What could help?
Is there anything else you would like to share? (regarding your expectations)

Table 2: interview guide for following interviews
Tell me how things are going for you.
Tell me about your feelings regarding the rehabilitation.
Is the rehabilitation going as you expected? Why/why not?
What thoughts do you have about your knee?
What is difficult right now?
What is easy right now?
What helps you the most right now?
Do you still have the same goal? If not, why?
What thoughts do you have about your sport?
